# Occupational hypersensitivity pneumonitis in a green tea manufacturer

**DOI:** 10.1002/rcr2.152

**Published:** 2016-03-31

**Authors:** Yuko Tanaka, Toshihiro Shirai, Noriyuki Enomoto, Kazuhiro Asada, Yoshiyuki Oyama, Takafumi Suda

**Affiliations:** ^1^ Department of Respiratory MedicineShizuoka General HospitalShizuokaJapan; ^2^ Second Department of Internal MedicineHamamatsu University School of MedicineHamamatsuJapan

**Keywords:** Granuloma, green tea, hypersensitivity pneumonitis, occupation

## Abstract

Hypersensitivity pneumonitis (HP) is caused by numerous agents, and one of its histopathological features is poorly formed granulomas. We report here a rare case of occupational HP caused by green tea, showing well‐formed granulomas. The patient, a 54‐year‐old woman who had worked for 15 years in a green tea factory, was referred for abnormal chest X‐ray shadows with cough and breathlessness over a 2‐month period. The chest X‐ray and high‐resolution computed tomography showed diffuse bilateral ground‐glass opacities and poorly defined centrilobular nodules. Histopathological examination of the thoracoscopic lung biopsy specimens showed bronchiolocentric interstitial pneumonia with well‐formed granulomas. Although the form of granulomas were atypical, laboratory data, CT findings, and intradermal skin testing suggested the diagnosis of subacute HP caused by green tea. After transfer to a different department, her condition improved markedly. Taking a precise medical history and avoidance of the suspected environmental agent proved useful in diagnosing this condition. © 2016 The Authors. *Respirology Case Reports* published by John Wiley & Sons Australia, Ltd on behalf of The Asian Pacific Society of Respirology

## Introduction

Hypersensitivity pneumonitis (HP) results from inhalation exposure to various types of agents, including microorganisms, dusts, aerosols, and chemicals, with histopathological features, including poorly formed granulomas 1, 2. Although very rare, green tea is reported as a causative agent of occupational HP 3, 4. However, the clinical course and histopathologic features of green tea‐induced HP are not fully understood. We present here a case of occupational HP in a green tea manufacturer, showing a subacute clinical course even after a long‐term exposure, and well‐formed granulomas by histopathological examination of the thoracoscopic lung biopsy specimens.

## Case Report

**Figure 1:**
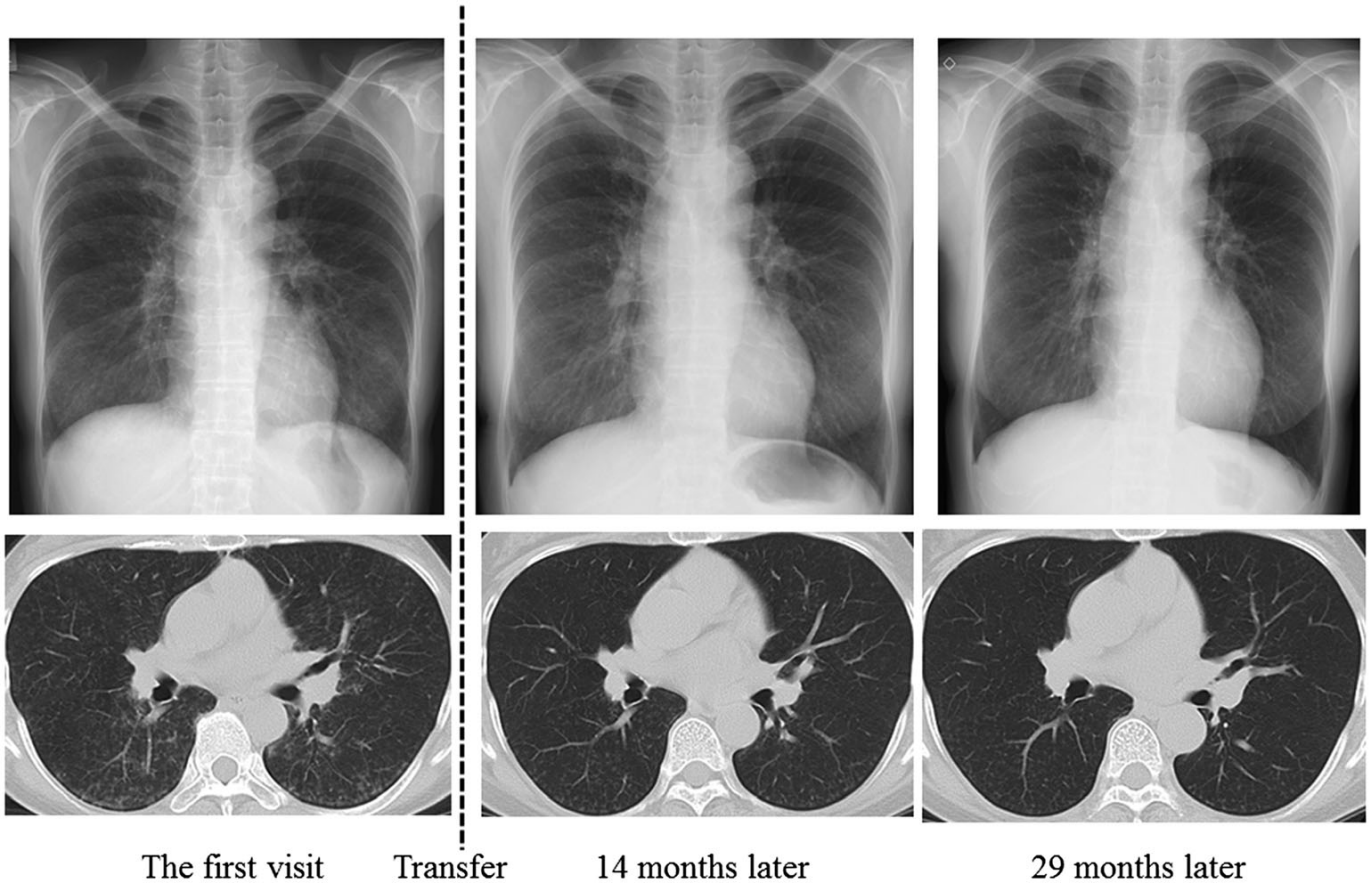
Chest X‐rays and high‐resolution computed tomography images on the first visit showed diffuse bilateral ground‐glass opacities and poorly defined centrilobular nodules. At 14 and 29 months after transfer to a different department of the factory, the images showed marked improvement.

**Figure 2:**
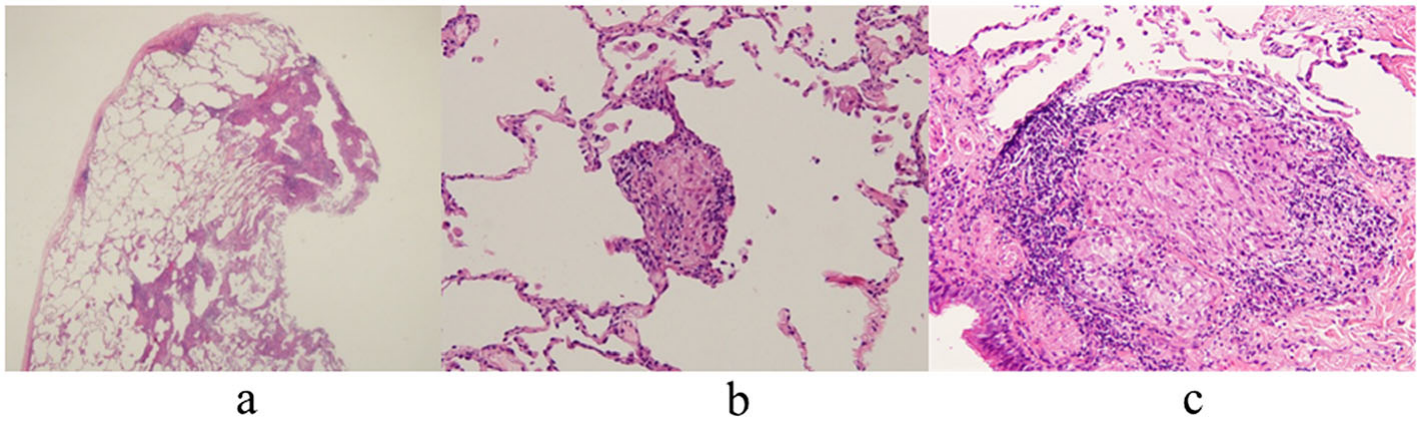
Histopathology showed cellular bronchiolitis with bronchiolocentric interstitial pneumonia (a). Some of the granulomas in the pulmonary interstitium were slightly larger and more well‐formed than those typically seen in hypersensitivity pneumonitis (b, c).

The patient, a 54‐year‐old woman with no history of smoking, had worked for more than 15 years without wearing a mask in a green tea factory. The work was to process tea leaves by using tea leaf‐processing machines. Although the dust concentration in the factory was not measured, the surface of the machines were covered with green tea dust, and floating dust in the air could be seen with the naked eye. Thus, she was thought to be exposed to a large amount of green tea dust. At the age of 50, she was diagnosed with sarcoidosis based on ocular symptoms and a high percentage of lymphocytes in bronchoalveolar lavage fluid (BALF) with normal chest X‐rays at a local hospital. She had no respiratory symptoms, and the results of transbronchial lung biopsy were nonspecific. Because her ocular symptoms were temporal and the ophthalmological examination was normal, she was followed up without any treatment. After 4 years, she was referred for abnormal chest X‐ray shadows with cough and breathlessness over a 2‐month period. Bronchoscopy, with bronchoalveolar lavage and transbronchial biopsies, was performed again at the local hospital. The cell differentiation of BALF demonstrated lymphocytosis (51.5%) with a predominance of CD8 *T*‐cells (CD4/CD8 0.4). Microscopic examination showed alveolitis with mainly lymphocytes. Because the diagnosis was uncertain, she was transferred to our hospital for further examinations. The chest X‐ray and high‐resolution computed tomography (HRCT) on the first visit showed diffuse bilateral ground‐glass opacities and poorly defined centrilobular nodules (Fig. 1). Fine crackles were audible over the posterior in both lungs. Laboratory data revealed KL‐6 775 U/mL, SP‐D 137 ng/mL, and serum antibody for *Trichosporon asahii*; the causative microorganism for summer‐type HP was negative. Pulmonary function tests showed a restrictive ventilatory impairment (FVC 2.01 L, %FVC 75.3%) and reduced diffusing capacity of the lung for carbon monoxide (DLCO 8.13 mL/min/mmHg, %DLCO 49.8%). A video‐assisted thoracoscopic lung biopsy was performed from right S10 to confirm the diagnosis. Histopathological examinations revealed cellular bronchiolitis with bronchiolocentric interstitial pneumonia. Some of the non‐necrotizing granulomas were slightly larger and more well‐formed than those typically seen in HP, which were located in the alveolar spaces, pulmonary interstitium, and pleura (Fig. 2). Intradermal skin tests with epigallocatechin gallate, the major soluble component of green tea leaves and the major causative agent of occupational green tea‐induced asthma 5, were performed. The early phase reaction (15 min) was negative, whereas the late phase reactions (6, 24, and 48 h) were positive. Although the granulomas were atypical, laboratory data, CT findings, and intradermal skin test results suggested a diagnosis of the subacute form of HP rather than sarcoidosis. Then, she was transferred to a different department of the factory with less exposure to green tea dust, and her symptoms were alleviated after 1 month and disappeared after 3 months. After 1 year, radiographic findings improved markedly (Fig. 1), laboratory data revealed KL‐6 438 U/mL and SP‐D 73.5 ng/mL, and pulmonary function tests showed normal values: FVC 2.74 L, %FVC 103.8 %, DLCO 14.58 mL/min/mmHg, and %DLCO 87.4%. Although other tests to exclude other causes of HP, that is, fungi were not done, the results of intradermal skin test and environmental avoidance strongly suggested that the green tea dust was the causative agent. Taking a precise medical history and avoidance of the suspected environmental agent proved to be useful in diagnosing this condition

## Discussion

We found two important clinical issues. First, occupational HP associated with green tea can present well‐formed granulomas in histopathology. Previous investigators reported that the histopathologic changes of HP included cellular bronchiolitis, interstitial pneumonia, and poorly formed non‐necrotizing granulomas located in the pulmonary interstitium 1, 2. A close study of HP granulomas at the Mayo Clinic revealed that well‐formed granulomas were present in 12.5% of patients 2. Among cases with granulomatous inflammation, granulomas are frequently encountered in the pulmonary interstitium (81%), alveolar spaces (85%), and pleural (9%) 2. Another study showed that farmer's lung tended to show granulomas, whereas bird fancier's lung tended to show no granuloma 1. To the best of our knowledge, there are only two case reports of HP associated with green tea, but neither investigated the presence of granulomas precisely 3, 4. In this case, the granulomas were well‐formed and located in the pulmonary interstitium, alveolar spaces, and pleura. It is likely that this is the first case report of HP induced by green tea showing well‐formed granulomas. One possibility is that there may be some association of the antigen and the histopathology of HP. Additionally, the clinical course of occupational HP associated with green tea can be regarded as subacute even after long‐term exposure. Otera *et al*. reported a case of a 51‐year‐old man who developed subacute HP after exposure to a high load of green tea components in the form of catechin inhalation 4. However, Sano *et al*. reported a case of a 54‐year‐old woman with subacute HP caused by green tea. In that case, the patient had been engaged in making tea bags for 18 years 3. In the present case, the patient had worked for more than 15 years in a green tea factory. Abnormal chest shadows with coughs and breathlessness occurred over a 2‐month period. In both cases, their symptoms improved markedly after transfer to another department in the factory. Although acute high load exposure in the form of cathecin inhalation causes subacute HP 4, these findings suggest that the subacute form of HP can occur even after long‐term exposure to the causal agent. The relationship between the mode of exposure (acute or chronic) and the form of HP development (acute, subacute, or chronic) does not always seem to be uniform.

## Disclosure Statements

No conflict of interest declared.

Appropriate written informed consent was obtained for publication of this case report and accompanying images.
